# Flexible Transparent Electrode of Hybrid Ag-Nanowire/Reduced-Graphene-Oxide Thin Film on PET Substrate Prepared Using H2/Ar Low-Damage Plasma

**DOI:** 10.3390/polym9010028

**Published:** 2017-01-13

**Authors:** Chi-Hsien Huang, Yin-Yin Wang, Tsung-Han Lu, Yen-Cheng Li

**Affiliations:** 1 Department of Materials Engineering, Ming Chi University of Technology, 84 Gungjuan Rd., Taishan Dist., New Taipei City 243, Taiwan; U99187001@gmail.com (Y.-Y.W.); U00187013@mail2.mcut.edu.tw (T.-H.L.); 2 Material and Chemical Research Laboratories, Industrial Technology Research Institute, 195, Sec. 4, Chung Hsin Rd., Chutung, Hsinchu 300, Taiwan; yorkli@itri.org.tw

**Keywords:** low damage plasma, reduced graphene oxide, flexible transparent electrode

## Abstract

We employ H_2_/Ar low-damage plasma treatment (H_2_/Ar-LDPT) to reduce graphene oxide (GO) coating on a polymer substrate—polyethylene terephthalate (PET)—with the assistance of atomic hydrogen (H_α_) at low temperature of 70 °C. Four-point probing and ultraviolet-visible (UV-Vis) spectroscopy demonstrate that the conductivity and transmittance can be controlled by varying the H_2_/Ar flow rate, treatment time, and radio-frequency (RF) power. Optical emission spectroscopy reveals that the H_α_ intensity depends on these processing parameters, which influence the removal of oxidative functional groups (confirmed via X-ray photoelectron spectroscopy) to yield reduced GO (rGO). To further improve the conductivity while maintaining high transmittance, we introduce silver nanowires (AgNWs) between rGO and a PET substrate to obtain a hybrid rGO/AgNWs/PET with a sheet resistance of ~100 Ω/sq and 81% transmittance. In addition, the hybrid rGO/AgNWs thin film also shows high flexibility and durability and is suitable for flexible and wearable electronics applications.

## 1. Introduction

Polyethylene terephthalate (PET) is a thermoplastic polyester resin and widely used in food and packaging industries [1,2]. Recently, the demands for wearable and flexible electronics applications, such as solar cells and displays are drastically increasing, PET is becoming a favorite polymer for such electronics applications due to its flexibility, light weight, high transparency and low cost. For the electronics applications, indium tin oxide (ITO), a transparent conductive oxide (TCO), is deposited onto PET substrate commercially as a transparent electrode owing to its conductivity and transparency in the visible range. However, ITO has some drawbacks, including high production costs, supply shortage and brittleness, which would restrict its application in flexible and wearable electronics.

As a result of its excellent optical transparency, flexibility and electrical conductivity, graphene has attracted research attention related to the development of transparent electrodes on flexible substrates. Such devices can be employed in various applications, including solar cells, touch panels, organic light emitting diodes, and wearable electronics [3,4,5,6]. Many graphene preparation methods have been developed, including micromechanical exfoliation of highly oriented pyrolytic graphite [7], sublimation of silicon from silicon carbide [8], and chemical vapor deposition from a carbon-containing gas [9].

Graphene oxide (GO), a derivative of graphene, is a promising alternative for the mass production of graphene-based materials because it can be synthesized in large quantities from inexpensive graphite and is soluble in various solvents. The preparation of a dispersed form of graphene for use in flexible electronic devices is attractive because this material is low cost and solution-processable. However, GO is almost insulating, due to the presence of oxidative functional groups. Therefore, the removal of these groups is necessary in order to convert GO into reduced graphene oxide (rGO). Typically, a chemical method with various reducing agents such as hydrazine, sodium borohydride, and hydroiodic acid, along with vitamin C, glucose, and alkali, is used to reduce GO [10,11,12,13,14,15]. However, chemical reducing agents are harmful to the environment and enable unexpected introduction of heteroatoms into rGO. In addition, such chemical methods are time-consuming and generally ineffective. Further, an additional post-annealing process at 500–1100 °C is required [16,17,18]. Although an annealing process in an Ar or H_2_ environment has been demonstrated to be an effective reduction method [19,20], the flexible PET substrate cannot withstand such high temperature.

Compared with the methods previously reported in the literature, plasma is environmentally friendly and has a short reducing time as part of a low-temperature process with a high-purity environment. For GO reduction, the generation of atomic hydrogen (H_α_) enables effective removal of the oxidative functional groups. However, it has been reported that both the ions and vacuum ultraviolet (VUV) radiation in plasma can break the graphene lattice and destroy the honeycomb-like carbon (C) nanostructure with ease, because their energies are higher than the C–C bond energies of *sp*^2^-hybridized C atoms in graphene [21,22,23,24]. As a result, the etching rate is generally too fast to allow precise control over the degree of reduction. Although downstream plasma treatment can be performed to reduce GO without ion bombardment, the resultant material remains sensitive to VUV radiation [25]. The high-intensity VUV radiation generated in a plasma system can greatly enhance the etching rate [26].

In our previous studies, we developed a low-damage plasma treatment (LDPT) method for the functionalization of nanocrystalline and thin-film materials for applications in flash memory devices and ion sensors, respectively [27,28]. Both results demonstrated successful functionalization with minimal plasma damage. By inserting a complementary filter into a parallel-plate plasma system, the ions and higher-energy VUV can be efficiently blocked. Thus, only radicals with extremely low kinetic energy, which have the highest reactivity among the plasma-generated species, can diffuse through the filter and reach the nanomaterials, gently functionalizing them. We have also employed this apparatus to oxidize graphene sheets without ions or VUV radiation; this oxidization could be conducted in a highly controllable manner [29,30]. Although Lee et al. reported remote hydrogen plasma at atmospheric pressure to avoid ion bombardment, in-situ annealing was required to obtain low sheet resistance of rGO [31]. In addition, utilization of hydrogen gas at atmospheric pressure is not a safe process.

In this study, an H_2_/Ar mixture gas is introduced into the LDPT apparatus, because the hydrogen radicals can be enhanced by Ar via penning ionization [32]. We systematically investigate the sheet resistance and transmittance of the obtained rGO, which is coated on a polyethylene terephthalate (PET) substrate, as functions of the H_2_/Ar ratio, treatment time, and plasma power. It is reported that silver nanowires (AgNWs) networks, which can be also prepared by low-cost solution-based process compatible with rGO preparation, exhibit high conductivity, high optical transmittance, and good mechanical flexibility [33,34]. However the large roughness of AgNWs and easy oxidation inhibit their applications [35,36]. In this study, we also investigated the introduction of AgNWs between the flexible substrate and rGO, so as to fabricate hybrid rGO/AgNWs thin films. In such a sample structure, the rGO can drastically lower the roughness of AgNWs, and the overall conductivity can be greatly improved while maintaining high transparency.

## 2. Materials and Methods

### 2.1. Sample Preparation

GO solution was purchased from Graphenea Inc., San Sebastián, Spain. The GO was dispersed in deionized (DI) water with a 4-mg/mL concentration. The GO was diluted using isopropanol (IPA) and then coated on an optical grade PET substrate (Nan Ya Plastics, BH215, New Taipei City, Taiwan) via spin-coating with 1000-rpm spin speed for 30 s to form GO sheets. Hereafter, these prepared samples are labeled “GO/PET.” Subsequently, the GO/PET was baked to remove the solvent thoroughly using a hot plate at 60 °C for 10 min. The thickness of as-prepared GO film was about 30 nm measured by atomic force microscope. The details were described in Supplementary Materials Figure S1. In addition, AgNWs were purchased from Sigma-Aldrich, Saint Louis, MO, USA (average diameter: 35 μm; length: 115 μm). The AgNWs were dispersed in IPA with a 0.5 wt % concentration. Before being coated onto PET substrate via drop casting, which was followed by drying at 60 °C for 30 min, the AgNWs were also diluted in IPA at various concentrations. This AgNW/IPA solution was then coated on the PET followed by the GO coating under the same conditions as those used for the GO/PET samples. The prepared samples, hereafter, are referred to as GO/AgNWs/PETs.

### 2.2. Low-Damage H_2_/Ar Plasma Treatment (H_2_/Ar-LDPT)

The reduction process to form rGO/PETs was performed using H_2_/Ar-LDPT. The plasma of LDPT was generated by an inductively coupled plasma system (see Supplementary Materials Figure S2a). The GO/PET samples were loaded onto the lower electrode beneath the grounded complementary filter and at a distance of 2 cm. The filter consists of upper and lower plates separated by a spacer (see Supplementary Materials Figure S2b). Both plates contain many stripes and slits of the same width. The slits on the upper plate are aligned with the stripes on the lower plate, which can block high-energy collimated ions and prevent direct exposure of the samples to UV radiation. In our previous study [27,28], we confirmed that the UV intensity can be reduced by more than 95%. Therefore, the filter can efficiently shield the samples from plasma damage caused by energetic ions and UV radiation. The filter only permits neutral and reactive radicals to diffuse through and reach the heated substrate, thus realizing a low-damage treatment process. The processing conditions included plasma generation via radio-frequency (RF) power, a 70 °C substrate temperature, and 0.3-Torr pressure. The H_2_/Ar ratio, treatment time, and RF power were varied in order to investigate their GO reduction ability. The generation of H_α_ in the plasma was confirmed via optical emission spectroscopy (OES), which was conducted through the vacuum-chamber view port. Note that OES is a powerful instrument for characterization of the excited states in a plasma environment.

### 2.3. Characterization of rGO and Hybrid rGO/AgNW Thin Films

After the rGO sheet preparation using H_2_/Ar-LDPT, the sheet resistance and transmittance were investigated using a four-point probe and ultraviolet-visible (UV-Vis) spectroscopy (V-670 UV-Vis-NIR spectrophotometer, JASCO, Tokyo, Japan). The transmittance of the PET substrate used in this study was 90%. The chemical compositions of the rGO sheets were examined using X-ray photoelectron spectroscopy (XPS; VG ESCA Scientific Theta Probe, Waltham, MA, USA) with a monochromated Al Kα source. Raman spectra were collected using a Horiba Raman system (iHR-550, Kyoto, Japan) with a laser wavelength of 532 nm to investigate the structure of rGO sheets. The Si peak at 520 cm^−1^ was used as the reference for wavenumber calibration prior to each measurement. A field emission scanning electron microscope (FESEM; JEOL JSM 6701F, Tokyo, Japan) and atomic force microscope (AFM; BRUCKER Dimension Edge, Billerica, MA, USA) were used to study the surface morphologies of AgNW and hybrid thin film.

## 3. Results and Discussion

**Figure 1:**
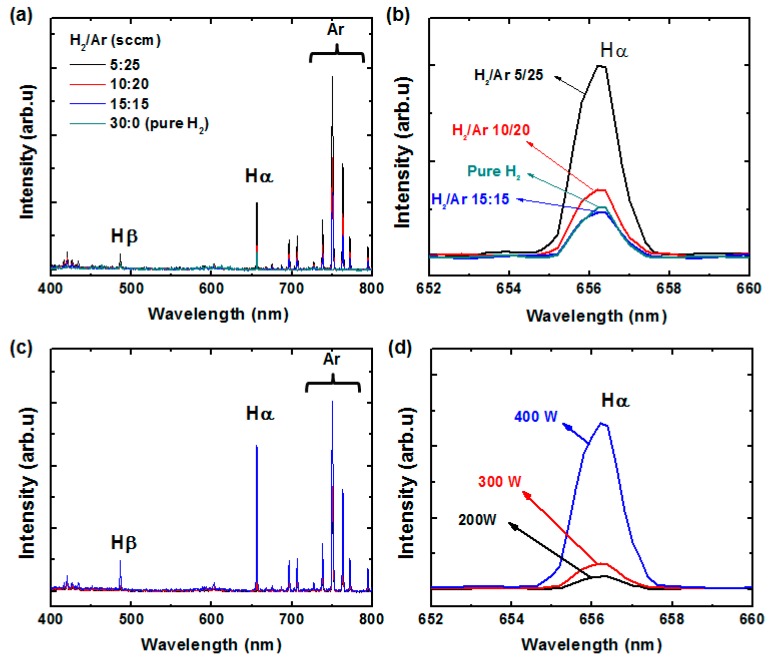
Optical emission spectra of H_2_/Ar plasma as functions of H_2_/Ar ratio at 300-W radio-frequency (RF) power (**a**,**b**) and RF power at H_2_/Ar ratio of 10/20 (**c**,**d**).

Figure 1a shows the H_2_/Ar plasma spectra obtained for various H_2_/Ar ratios using OES. For all spectra, dense lines corresponding to Ar can be observed in the 700–800 nm range. A peak is positioned at 656.3 nm for all H_2_/Ar mixing ratios, which is due to the presence of H_α_. From Figure 1b, as the Ar flow rate increases, the H_α_ intensity increases; this change results from penning ionization [31]. When the Ar flow rate is decreased, corresponding to a H_2_/Ar ratio of 15/15, the H_α_ intensity is comparable to that of pure H_2_ plasma, indicating reduced penning ionization. Accordingly, in what follows, we neglect the pure H_2_ plasma conditions affecting the rGO characteristics. Figure 1c shows the H_2_/Ar plasma spectra obtained for various RF powers and a fixed H_2_/Ar ratio of 10/20. The relative H_α_ intensity was found to increase with increased RF power as shown in Figure 1d. From the OES results, it is clearly apparent that the H_2_/Ar ratio and RF power greatly influence the H_α_ intensity, which is the main species that reduces GO.

**Figure 2:**
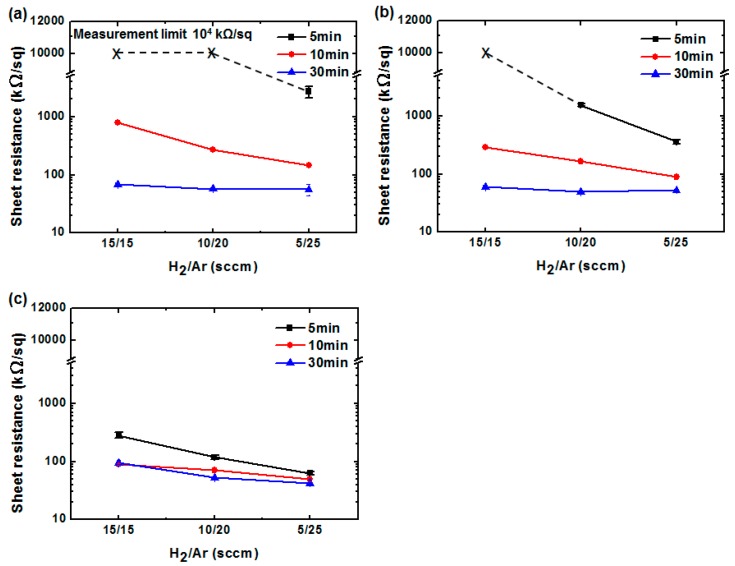
Reduced graphene oxide (rGO) sheet resistance for various treatment times and H_2_/Ar ratios at RF powers of: (**a**) 200; (**b**) 300; and (**c**) 400 W. (GO sheet resistance is unmeasurable).

Next, we systematically investigated the reduction effect on the GO conductivity and transmittance by varying the H_α_ intensity and treatment time. The as-coated GO sheet exhibited no conductivity and transmittance of 88.9% at wavelength of 550 nm. Figure 2a shows the sheet resistance trends obtained for different treatment times and H_2_/Ar ratios at an RF power of 200 W. For all treatment times, the H_2_/Ar ratio of 5/25 exhibits the lowest sheet resistance. As the Ar flow rate resistance decreases. In particular, in the case of the five-minute treatment time, all the sheet resistances obtained for H_2_/Ar ratios of 10/20 and 15/15 exceeded the measurement limitation (10^4^ kΩ/sq), as denoted by the “x” symbols in Figure 2a. This was because of the low H_α_ intensity, which prevented achievement of GO reduction within a short treatment time. In the case of 10-min treatment, the sheet resistance decreases slightly with increased Ar flow rate. This result also corresponds to decreased H_α_ intensity, as shown in Figure 1b. Furthermore, for the 30-min case, the sheet resistances remained at almost 60 kΩ/sq, regardless of the H_2_/Ar ratio. This result also suggests that the maximum reduction can be achieved for a sufficiently long treatment, even though the plasma exhibits the lowest H_α_ intensity under 200-W RF power. It is worth noting that the temperature did not increase during the 30-min treatment, because LDPT is a very low-damage treatment that efficiently eliminates the ion bombardment that induces a temperature increase. Figure 2b shows the sheet resistance depending on the treatment time and H_2_/Ar ratio at an RF power of 300 W. The result has almost the same trend as that observed in Figure 2a, except for the value obtained for the H_2_/Ar ratio of 10/20 and five-minute treatment. The sheet resistance of the rGO/PET sample under these conditions was measurable. For the 30-min case, the sheet resistance values also remained at the same level (ca. 50 kΩ/sq) as those obtained for 200-W RF power, for all H_2_/Ar ratios. Figure 2c shows the sheet resistance for different treatment times and H_2_/Ar ratios under 400-W RF power. For the five-minute case, the sheet resistance values decreased slightly with increasing Ar flow rate, due to the increase of H_α_. On the other hand, the sheet resistances for the 10- and 30-min cases are almost at the same level and are not related to the H_2_/Ar ratio. This result suggests that the reduction time can be shortened significantly when a high RF power is used. The prepared rGO sheets showed good stability by confirming the variation of sheets resistance as the samples were stored in atmosphere (see Supplementary Materials Figure S3).

**Figure 3:**
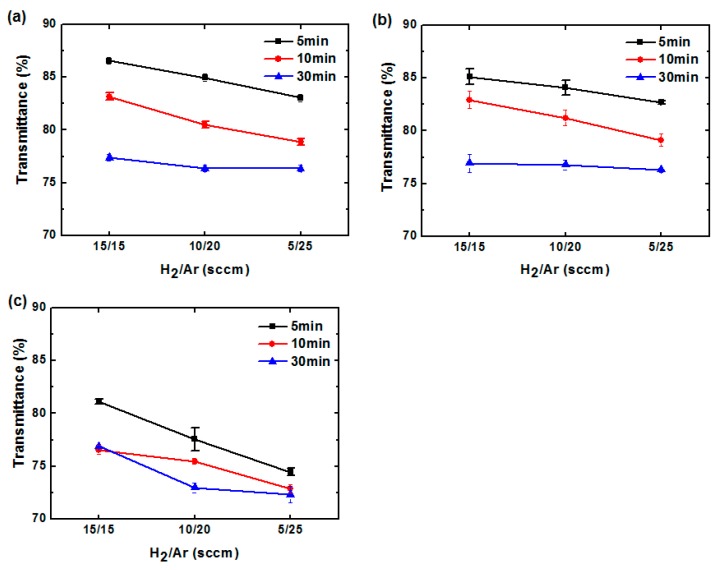
rGO transmittance (@ 550 nm) for various treatment times and H_2_/Ar ratios at RF powers of: (**a**) 200; (**b**) 300; and (**c**) 400 W. (GO transmittance @ 550 nm is 88.9%).

The transmittance spectra of GO sheets treated with different treatment times, H_2_/Ar ratios, and RF powers were recorded by UV-Vis spectrophotometer (see Supplementary Materials Figure S4). The transmittances at 550-nm wavelength for different treatment times and H_2_/Ar ratios under RF powers of 200, 300 and 400 W are displayed in Figure 3a–c, respectively. For 200-W RF power and all H_2_/Ar ratios, the transmittances of the rGO/PET samples exhibit low values with increased treatment time. It has been reported that GO absorption is high in the near-infrared range, and that graphene theoretically absorbs 2.3% of visible light at 550 nm [37,38]. Therefore, it is thought that a greater reduction corresponds to a lower transmittance. In other words, a trade-off must be made between conductivity and transmittance. In this study, the lowest transmittance values were observed for 400-W RF power (Figure 3c). The corresponding sheet resistance values were also the lowest.

**Figure 4:**
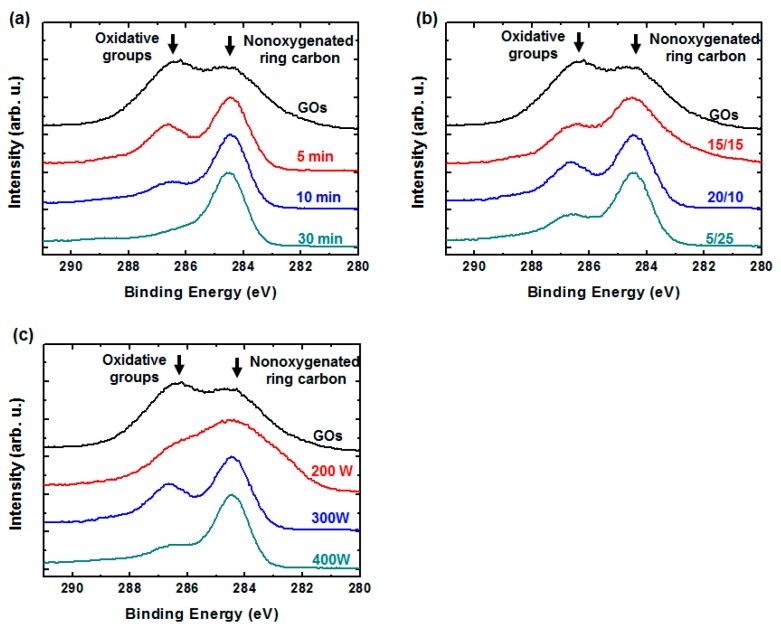
XPS C 1s spectra of rGO before and after H_2_/Ar-low-damage plasma treatment (H_2_/Ar-LDPT) as functions of: (**a**) H_2_/Ar ratio; (**b**) treatment time; and (**c**) RF power. Standard plasma conditions: RF power: 300 W; H_2_/Ar ratio: 10:20 (sccm); treatment time: five minutes.

The oxygen bonding in the rGO sheets after the H_2_/Ar-LDPT process was examined using XPS. Figure 4a–c show the treatment time, H_2_/Ar ratio, and RF power dependences of the C 1s spectra, respectively. In addition to nonoxygenated ring carbon (C–C) located at 284.5 eV, the XPS C 1s spectra of the GO sheets feature signals at higher chemical shifts for oxidative functional group, including hydroxyl groups (C–OH), carbonyl groups (C=O), and carboxyl groups (COOH) [29,39]. After the H_2_/Ar-LDPT process, the intensities of the oxidative functional groups located at the higher chemical shifts decreased, indicating GO reduction. It is also apparent that more oxidative functional groups were removed when the treatment time was longer as shown in Figure 4a. Figure 4b,c show the H_2_/Ar ratio and RF power dependency on the removal of the oxidative functional groups. As the Ar flow rate and RF power increased, more oxidative functional groups were removed, because of the higher H_α_ intensity. These results are consistent with those for the sheet resistance and H_α_ intensity, shown in Figure 1 and Figure 2, respectively, indicating that greater removal of the oxidative functional groups corresponds to a higher reduction level, which, in turn, leads to higher conductivity.

**Figure 5:**
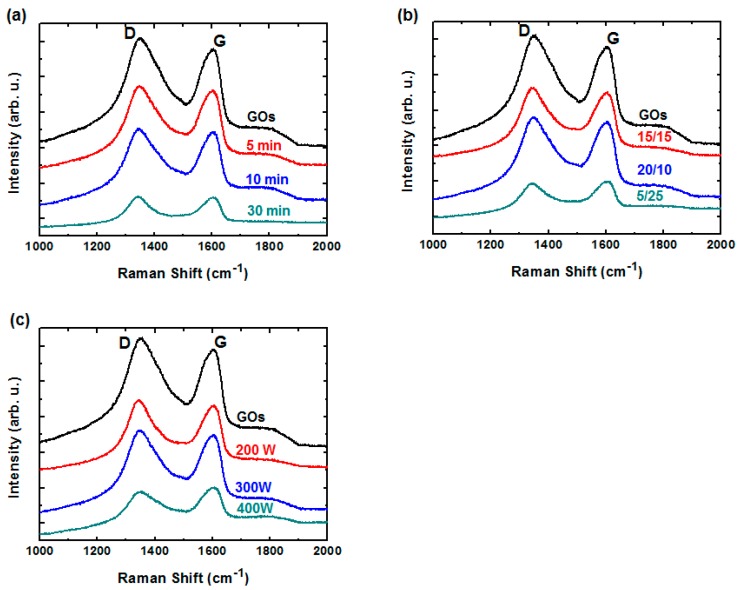
Raman spectra rGO sheets before and after H_2_/Ar-LDPT as functions of: (**a**) H_2_/Ar ratio; (**b**) treatment time; and (**c**) RF power. Standard plasma conditions: RF power: 300 W; H_2_/Ar ratio: 10:20 (sccm); treatment time: five minutes.

**Figure 6:**
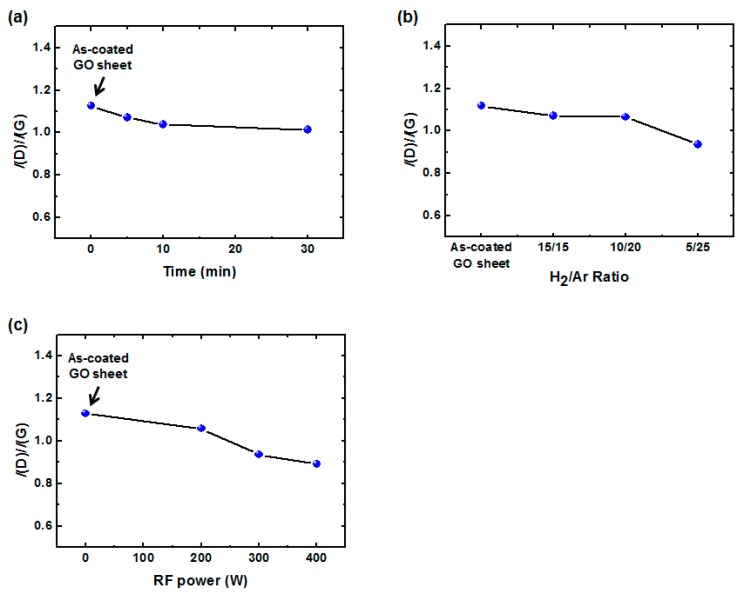
Intensity ratio of the D and G bands (*I*(D)/*I*(G)) of rGO sheets before and after H_2_/Ar-LDPT as functions of: (**a**) H_2_/Ar ratio; (**b**) treatment time; and (**c**) RF power. Standard plasma conditions: RF power: 300 W; H_2_/Ar ratio: 10:20 (sccm); treatment time: five minutes.

Following the characterization of chemical compositions of GO sheets as a function of various H_2_/Ar-LDPT process conditions, structural changes to the GO sheets resulting from H_2_/Ar-LDPT were investigated by Raman spectroscopy. Figure 5a–c displays the Raman spectra of GO sheets treated with different treatment times, H_2_/Ar ratio, and RF powers. The Raman spectrum of as-coated GO sheet shows a G-band (ca. 1600 cm^−1^) and D-band (ca. 1350 cm^−1^) corresponding to ordered sp^2^ bonded carbon and structural defects created by oxygenated functional groups, respectively [40]. After the H_2_/Ar-LDPT process, as shown in Figure 5a, the relative intensities of G and D slightly changes as the treatment time increases. The intensity ratio of the D and G bands (*I*(D)/*I*(G)) is commonly used as an index for the defect level existing in graphene. As exhibited in Figure 6a, the *I*(D)/*I*(G) of as-coated GO sheet is 1.13 and the ratio slightly decreased as the treatment time increased. The *I*(D)/*I*(G) is 1.01 for treatment time of 30 min. The dependencies of *I*(D)/*I*(G) on H_2_/Ar ratio and RF power are displayed in Figure 6b,c. As the Ar flow rate and RF power increased, *I*(D)/*I*(G) decreased prominently. The lowest values of *I*(D)/*I*(G) for Ar flow rate of 25 sccm and RF power of 400 W are 0.94 and 0.89, respectively. Those results indicated that the more the H_α_ generates in H_2_/Ar LDPT process, the slightly lower the defect level of rGO sheet is obtained. Moreover, it has been reported that reduction by hydrazine hydrate or hydroiodic acid leads to higher *I*(D)/*I*(G) corresponding to the generation of defects [8,9,10]. The decrease in *I*(D)/*I*(G) of GO sheets treated by H_2_/Ar-LDPT indicates that the reduction process slightly repairs the flakes of GO sheets without introducing additional defect due to plasma damage.

**Figure 7:**
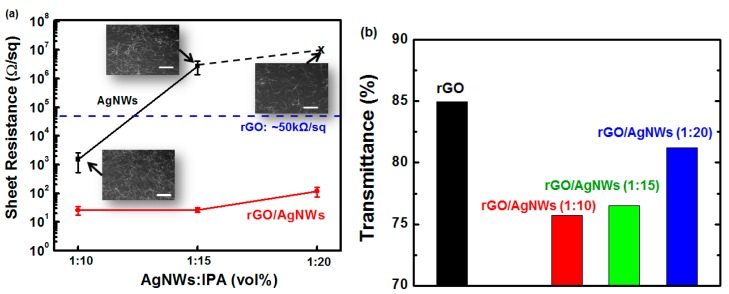
(**a**) Sheet resistance and (**b**) transmittance of silver nanowires (AgNWs) and hybrid rGO/AgNWs thin films as functions of AgNW concentration. Insets: SEM images of AgNWs with various solvent concentrations. Scale bar: 1000 μm.

In order to further reduce the sheet resistance of the rGO/PET samples while maintaining the relatively high transmittance, we introduced AgNWs between the GO and PET before the H_2_/Ar-LDPT process. The process conditions used to obtain hybrid rGO/AgNWs thin films on the PET were as follows: RF power: 300 W; H_2_/Ar ratio: 10/20; treatment time: five minutes. The AgNWs were diluted in IPA in three ratios (AgNWs:IPA): 1:10, 1:15, and 1:20. Figure 7a shows the sheet resistance values of the AgNWs with various dilution ratios. As the diluted solvent (isopropanol (IPA)) content increased, the sheet resistance increased. For the AgNW/IPA ratio of 1:20, the sheet resistance exceeded the measurement limitation. The insets of Figure 7a are SEM images of the AgNW thin films. As the IPA content increased, the AgNW network became coarse, leading to poor conductivity as the connecting points between each AgNW were reduced. After spin-coating GO on the AgNWs and then subjecting them to H_2_/Ar-LDPT, the sheet resistance values of the resultant rGO/AgNWs/PET samples were almost identical. This result indicates that the AgNWs were embedded into the rGO during spin-coating, which compensated for the discontinuity between each AgNW and yielded considerable enhancement in the conductivity. The transmittance spectra were measured by UV-Vis spectrophotometer (see Supplementary Materials Figure S5). Figure 7b shows the transmittance values of the various rGO/AgNWs/PET samples at wavelength of 550 nm. The sample with the 1:20 dilution ratio exhibited the highest transmittance of 81%. Thus, we obtained hybrid rGO/AgNWs thin films on a flexible PET substrate with high conductivity (~100 Ω/sq) and high transmittance (~81%).

**Figure 8:**
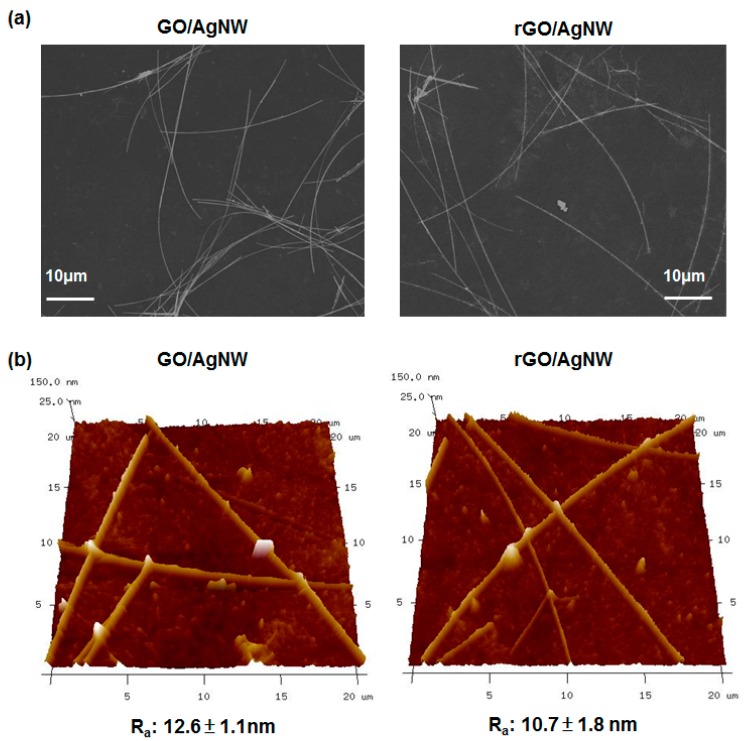
(**a**) SEM and (**b**) AFM images of hybrid thin films before (GO/AgNW) and after (rGO/AgNW) H_2_/Ar-LDPT.

To investigate the influence of H_2_/Ar-LDPT process on the morphology of the hybrid thin film, SEM and AFM measurements were conducted. Figure 8a shows the top-viewed SEM images of hybrid thin films before (GO/AgNWs) and after (rGO/AgNWs) H_2_/Ar-LDPT process, respectively. No obvious difference was observed. Figure 8b shows the AFM images of hybrid thin films before and after H_2_/Ar-LDPT process, respectively. The roughness (*R*_a_) of the hybrid thin film was slightly decreased after H_2_/Ar-LDPT process.

**Figure 9:**
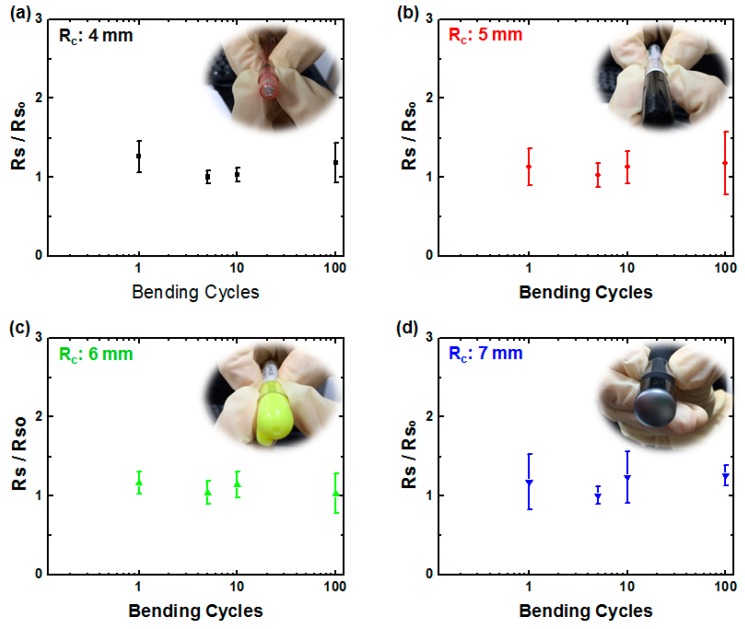
Flexibility test of hybrid rGO/AgNWs thin film (AgNW:isopropanol (IPA) = 1:20) under various radii of curvature (*R*_c_): (**a**) 4 nm; (**b**) 5 nm; (**c**) 6 nm and (**d**) 7 nm. Insets show the photos of cylindrical objects with various radius *R*.

Finally, the mechanical flexibilities of rGO/AgNW/PET samples were studied by placing them on cylindrical objects with various radii of curvature (*R*_c_). The *R*_c_ was estimated by measuring the outer diameter of the objects. In this measurement, the samples were bent to wrap various cylindrical objects, including battery, pen and laser-pointer, and then relaxed for bending cycles of 1, 5, 10 and 100. After each bending cycle, the sheet resistance of rGO/AgNWs/PET was measured. Figure 9 shows the results of sheet resistances of rGO/AgNWs/PET samples after each bending cycles depending on various *R*_c_s of cylindrical objects expressed by *R*s/*R*s_0_, where *R*s and *R*s_0_ are sheet resistances of before and after bending, respectively. The insets show the photos of rGO/AgNWs/PET wrapping on cylindrical objects with various *R*_c_s. It is clearly seen that the *R*s/*R*s_0_ shows no apparent dependence on bending cycle or *R*_c_, and all the values only slightly increases above 1. The results indicate that the morphology of the network of rGOs and AgNWs in hybrid rGO/AgNWs thin film was maintained during the bending test, demonstrating high flexibility and durability.

## Supplementary Materials

The supplementary materials are available online at www.mdpi.com/2073-4360/9/1/28/s1.
