# Supplementary material for: Flexible Transparent Electrode of Hybrid Ag-Nanowire/Reduced-Graphene-Oxide Thin Film on PET Substrate Prepared Using H2/Ar Low-Damage Plasma
Source: Polymers (Basel). 2017 Jan 13;9(1):28. doi: 10.3390/polym9010028 (PMC6431900; doi:10.3390/polym9010028)
Supplement: Supplementary file 1 [file polymers-09-00028-s001.pdf]

Supplementary Materials: Flexible Transparent Electrode of Hybrid Ag-Nanowire/Reduced-Graphene-Oxide Thin Film on PET Substrate Prepared Using H2/Ar Low-Damage Plasma

Chi-Hsien Huang, Yin-Yin Wang, Tsung-Han Lu and Yen-Cheng Li

In order to measure the thickness of GO film, we put kapton tape on PET substrate, where no GO could be coated on, during GO spin-coating. After baking at 60 °C for 10 min, the kapton tape was removed to create a boundary (the white dashed line in Figure S1a) where the height difference can be measured by atomic force microscope. Typical thickness of GO was shown in Figure S1b.

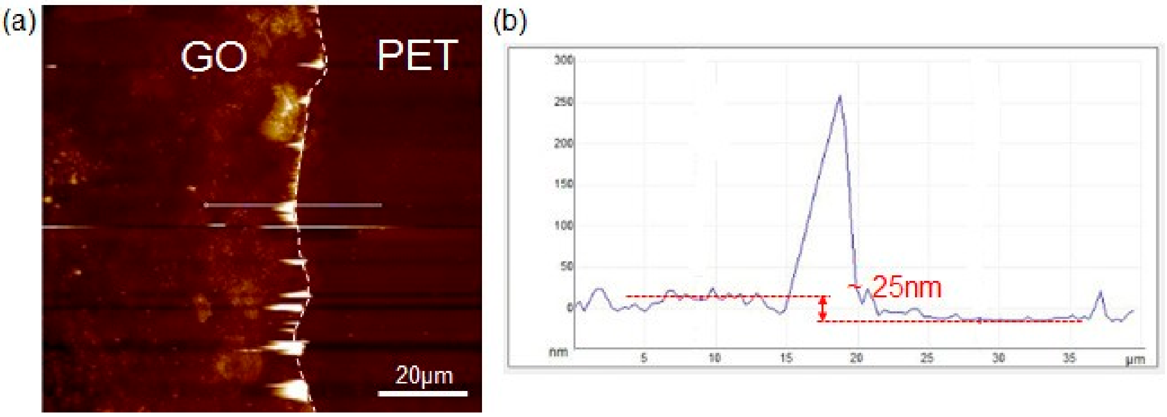

Figure S1. (**a**) Atomic force microscope image of GO film on PET substrate; (**b**) height difference between GO and substrate.

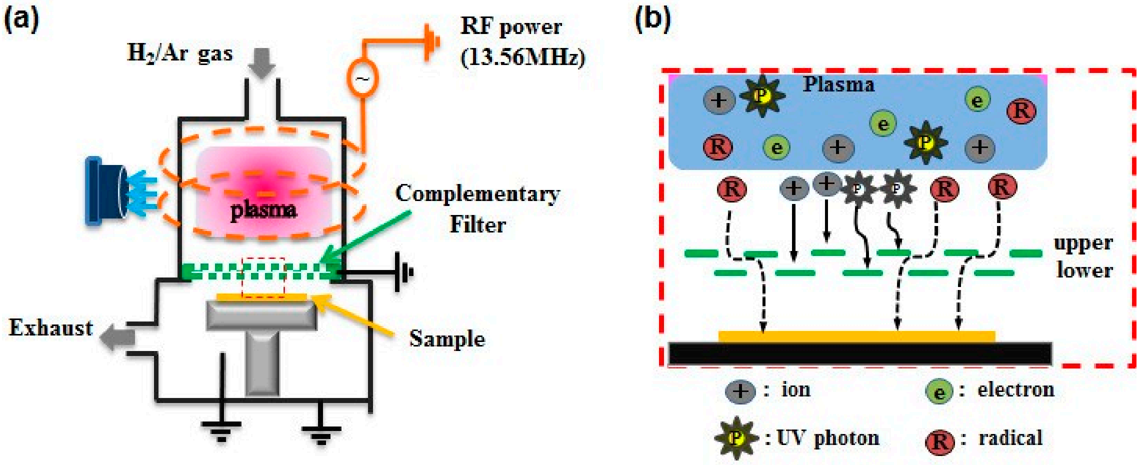

Figure S2. (**a**) Low damage plasma treatment with inductively coupled plasma source and a complementary filter and (**b**) enlargement of read dashed region of Figure S1a.

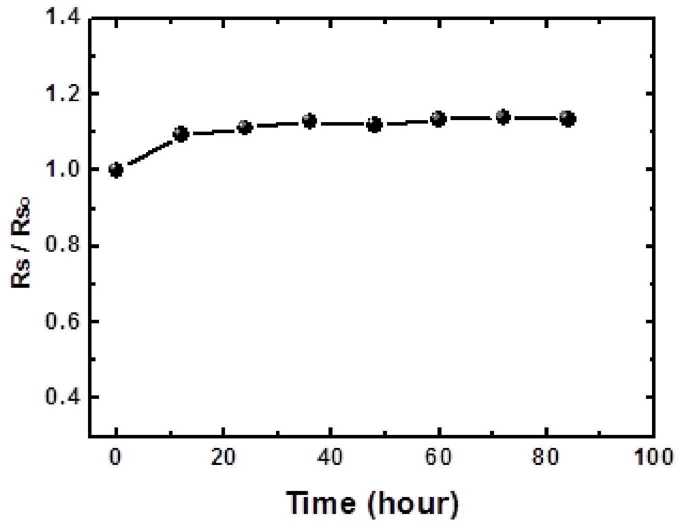

Figure S3. Stability of sheet resistance as the prepared rGO sheet exposed to atmosphere.

To investigate the stability of prepared rGO sheet when exposed to atmosphere, we measured the variation of sheets resistance every 12 h expressed by R_s/R_{s0}, where R_s and R_{s0} are sheet resistances of as-prepared sample and after exposing to atmosphere, respectively. The rGO sheet was prepared under the plasma conditions: RF power: 300 W; H_2/Ar ratio: 10/20; treatment time: 5 min. The sheet resistance slightly increased after 12 h and reach plateau indicating stable electrical property.

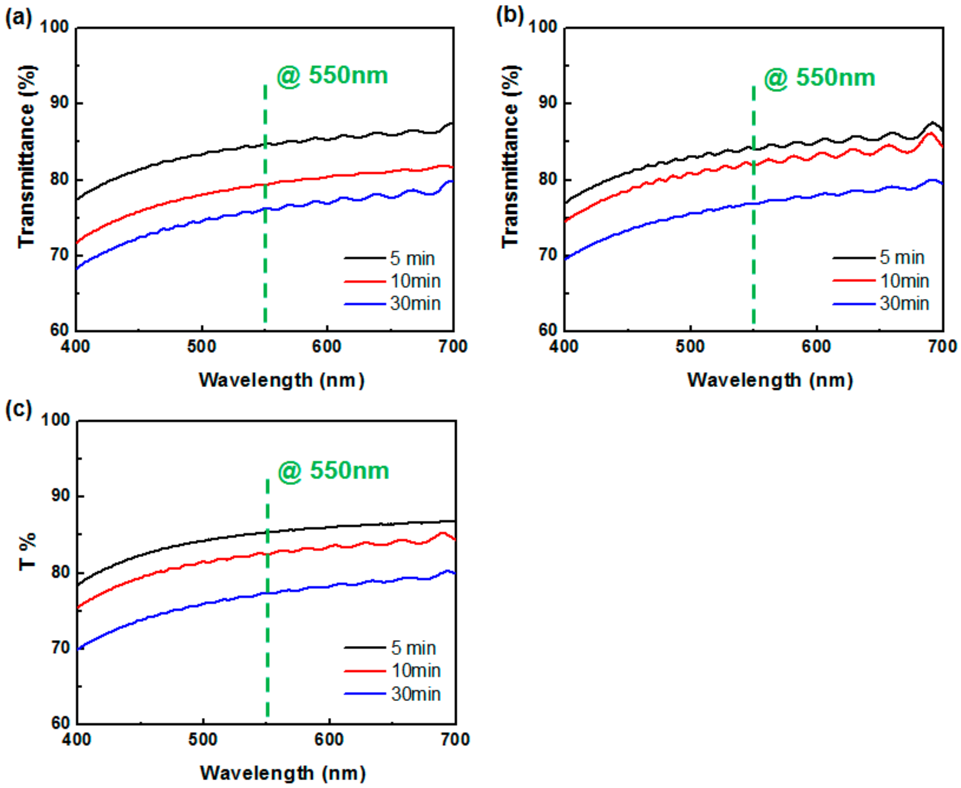

Figure S4. UV-Vis spectrum of rGOs for various H_2/Ar ratios of (**a**) 5/25; (**b**) 10/20; (**c**) 15/15 at treatment times of 5, 10 and 30 min and RF power of 200 W.

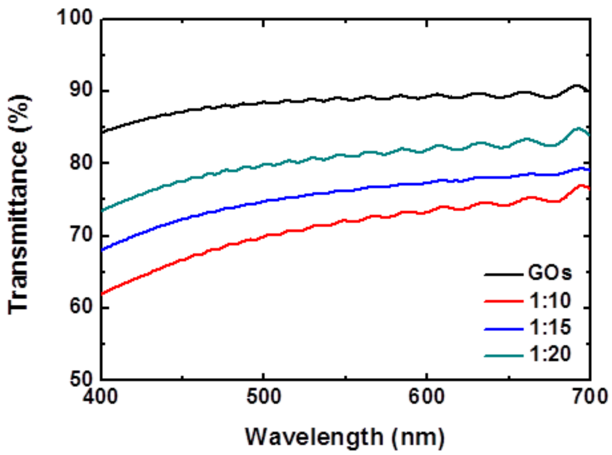

Figure S5. UV-Vis spectrum of AgNW/rGOs hybrid thin films as a function of AgNW concentration.
